# The Perspiration of Calcarea

**Published:** 1898-08

**Authors:** J. M. Selfridge

**Affiliations:** Oakland, Cal.


### THE PERSPIRATION OF CALCAREA.

OAKLAND, CAL., April 15th, 1898.

EDITOR OF THE HOMŒOPATHIC PHYSICIAN: You will be kind enough to pardon me for calling your attention to what I think is an error in your editorial in February number of THE HOMŒOPATHIC PHYSICIAN, page 50, seventh line from the top. You are comparing Silicea and Calcarea-carbonica. You say Calcarea has sour perspiration on the head.

Now, so far as my knowledge and observation goes, this is a mistake. I know that the symptoms of the two remedies are often very much alike, but this has been one of my key-notes as between the two. Silicea has sour sweat; Calc. has not. I know Calc. has sour secretions generally, but not so with perspiration.

If I am wrong I would be glad to be set right. Please tell me where you find it. Very respectfully,

J. M. SELFRIDGE.

[Dr. Selfridge is probably right. The symptom referred to stands as a note in the handwriting of the editor (made in 1868) to symptom nineteen of Silicea in Lippe's *Materia Medica*, page 627. It is not found elsewhere either in Lippe, Hering, Jahr, or Bœnninghausen, and may have been an error in reporting Dr. Lippe's words.—ED.]

